# Transthyretin: From Structural Stability to Osteoarticular and Cardiovascular Diseases

**DOI:** 10.3390/cells10071768

**Published:** 2021-07-13

**Authors:** Elżbieta Wieczorek, Andrzej Ożyhar

**Affiliations:** Department of Biochemistry, Molecular Biology and Biotechnology, Faculty of Chemistry, Wroclaw University of Science and Technology, Wybrzeże Wyspiańskiego 27, 50-370 Wroclaw, Poland; andrzej.ozyhar@pwr.edu.pl

**Keywords:** heart disease, atherosclerosis, thrombosis, inflammation, COVID-19, amyloid, biomineralization, ROS

## Abstract

Transthyretin (TTR) is a tetrameric protein transporting hormones in the plasma and brain, which has many other activities that have not been fully acknowledged. TTR is a positive indicator of nutrition status and is negatively correlated with inflammation. TTR is a neuroprotective and oxidative-stress-suppressing factor. The TTR structure is destabilized by mutations, oxidative modifications, aging, proteolysis, and metal cations, including Ca^2+^. Destabilized TTR molecules form amyloid deposits, resulting in senile and familial amyloidopathies. This review links structural stability of TTR with the environmental factors, particularly oxidative stress and Ca^2+^, and the processes involved in the pathogenesis of TTR-related diseases. The roles of TTR in biomineralization, calcification, and osteoarticular and cardiovascular diseases are broadly discussed. The association of TTR-related diseases and vascular and ligament tissue calcification with TTR levels and TTR structure is presented. It is indicated that unaggregated TTR and TTR amyloid are bound by vicious cycles, and that TTR may have an as yet undetermined role(s) at the crossroads of calcification, blood coagulation, and immune response.

## 1. TTR Structure and Major Functions

Human TTR (UniProtKB, P02766) is a homotetrameric, slightly acidic (isoelectric point of 5.3) protein with a molecular weight of 55 kDa (Protparam, https://web.expasy.org/protparam/) that binds and transports thyroid hormones and retinol in the plasma and cerebrospinal fluid (CSF) [1,2]. TTR is synthesized predominantly in the liver and choroid plexus [3,4]. However, TTR is also present in kidneys, the pineal gland, and in certain types of cells, such as neurons, retinal pigment epithelium, or pancreatic island cells [2,5,6,7,8,9]. TTR expression in the liver is controlled by hepatocyte nuclear factors [10]. In the brain, TTR synthesis is enhanced by heat shock factor 1 [11] and mitochondrial transcription factor A (TFAM) [12]. Stress and steroid hormones, phenylalanine, and its metabolites also regulate TTR expression [9,13,14]. The central ligand-binding channel is formed when four TTR monomers assemble into the complex [15]. This hydrophobic cavity is able to accommodate up to two thyroxine molecules. However, due to negative cooperativity, TTR carries only a single thyroxine molecule [16]. In plasma, human TTR binds, with intermediate affinity, 15–20% of the thyroid hormone pool, complementing the transport provided by thyroxine-binding globulin and albumin. However, TTR is the major thyroid hormone-binding protein in CSF [17,18]. This binding protects thyroid hormones from adsorption on lipid phases; therefore, almost all hormone molecules circulate in a protein-bound form [18]. On the other hand, only a small population of TTR molecules carry the ligand because TTR concentration greatly exceeds the thyroid hormone level in both plasma and CSF [17,19,20,21]. The central channel of TTR can also tolerate multiple other natural ligands, including lutein and other carotenoids, curcumin, polyphenols (resveratrol), norepinephrine oxidation products, and other compounds [1,22,23,24]. TTR also distributes retinol complexed with retinol-binding protein (RBP), and the binding stoichiometry is 1:1:1 [25]. RBP binding is able to influence physiological function of TTR. RBP abrogates the binding of soluble TTR to receptor for advanced glycation end products (RAGE) [26]. Aggregated (fibrillar) TTR activates nuclear factor κB through interaction with RAGE and induces inflammatory and apoptotic responses [26]. Additionally, the interaction of TTR with perlecan, a component of the basement membrane, is negatively influenced by RBP [27]. Approximately 1–2% of TTR molecules circulate in the plasma in association with high density lipoproteins (HDL) due to the binding to apolipoprotein A1 (ApoA1) [28]. However, the interaction of TTR with ApoA1 is not affected by RBP [28]. The neurogenic effects of TTR are also not dependent on its ligands: RBP and thyroxine [29]. TTR has neuroprotective functions and/or is a stress response factor in various adverse conditions, including ischemia, Alzheimer’s disease (AD), Crohn’s disease, osteoarthritis, and preeclampsia [29,30,31,32,33]. Interestingly, RBP, by stabilizing TTR structure, reduces the inhibitory effect of TTR on Aβ aggregation [34]. In addition to neuroprotection and neurogenic activity, TTR is engaged in insulin secretion, autophagy, memory, and behavior [35,36,37,38,39]. TTR is a diagnostic marker positively correlated with nutrition, for both protein and glucose metabolism, and negatively correlated with acute inflammatory states [40]. Importantly, TTR is a Zn^2+^-dependent protease (also active in the presence of Mn^2+^, Fe^3+^, and Co^2+^) that cleaves ApoA1, neuropeptide Y (NPY), and amyloid β (Aβ) peptide [41,42,43].

## 2. Structural Stability, Cleavage, and Amyloidogenesis of TTR

**Figure 1:**
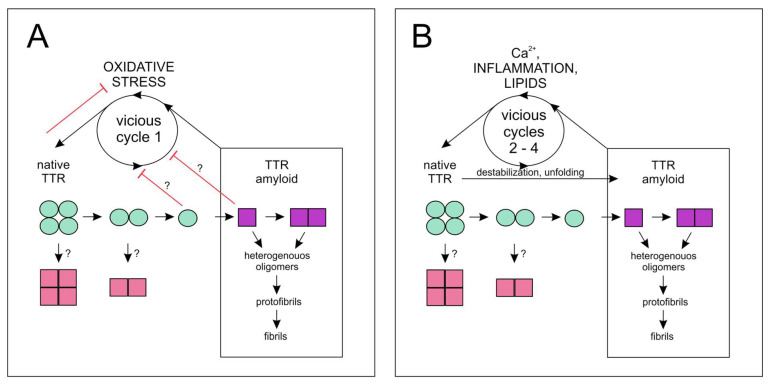
Amyloid formation pathways and vicious cycles of TTR. Models showing amyloid formation pathways and correlations between TTR form/stability and factors that affect TTR stability and drive the vicious cycles. Properly folded and partially unfolded forms of TTR are indicated by circles and squares, respectively. Native TTR tetramers dissociate to unstable dimers and then to monomers. The unfolded, aggregation-prone monomers dimerize, and then form heterogeneous populations of oligomers, some of which assemble into protofibrils and fibrils. Vicious cycle 1 (**A**) differs from other cycles (**B**) in the protective role of TTR against oxidative stress. Question marks denote the direct unfolding (from properly folded) of TTR tetramers and dimers, as well as the contribution of folded and unfolded monomeric forms of TTR in protection against oxidative stress, which requires further research.

The TTR monomer is composed of 127 amino acids and has a β-sandwich structure with immunoglobulin-like topology, with a rigid core composed of two β-sheets of four anti-parallel β-strands connected with flexible loops. A single short α-helix is located between the E and F strands [15,44]. A short (approximately ten amino acids) fragment of the N-terminus is disordered. The protective and physiological functions of TTR are dependent on the stability of the TTR structure [21,22]. TTR stability strongly relies on the hydrogen bonding network formed by intramolecular interactions with water molecules and intermolecular interactions with the solvent [45,46]. The flexibility of the loops (particularly the DE loop) is critical for TTR stability, and conformational fluctuations contribute to aggregation propensity of TTR [47,48,49,50,51]. The binding of thyroxine and some other ligands (resveratrol and curcumin) increases structural stability of TTR [21,22,52]. On the other hand, proper TTR structure is negatively affected by oxidative modifications, aging, metal cations (including Ca^2+^), and most of the known TTR mutations [21,47,53,54,55,56,57]. Destabilizing mutations negatively affect the association between TTR subunits, which leads to the dissociation of the tetramer to dimers, followed by their fast decomposition to monomers [57,58], as shown in Figure 1. The dissociation of TTR is connected to conformational alterations that result in partially unfolded monomers assembling into aggregates with diverse quaternary structures [49,51,59,60,61,62,63]. High molecular weight (HMW) oligomers are highly cytotoxic, dynamic, heterogeneous, and transiently populated species of TTR that represent intermediate states of early steps of amyloidogenesis [61,62,63,64]. TTR aggregates undergo structural rearrangements forming protofibrils and mature fibrils characterized by a new ordered cross-β structure [51,61,62,65]. The formation of TTR amyloid deposits of different morphologies underlies the pathogenesis of severe and potentially lethal diseases, i.e., amyloidoses [64,66,67]. Amyloid formation from aggregated wild-type TTR (ATTRwt) results in senile systemic amyloidosis (SSA), whereas deposits of aggregated TTR variants/mutants underlie familial amyloidotic polyneuropathy (FAP) and familial amyloidotic cardiomyopathy [66]. In the familial type of the disease, TTR depositions are localized predominantly extracellularly in the brain and heart tissues, and SSA depositions associated with age are detected in many organs, mainly in the heart, lungs, and blood vessels [51,66,67,68]. Amyloid depositions in SSA (and in some FAP) patients are extensive and diffused, and contain tightly packed, short, and randomly oriented fibrils (amyloid A) [66,67,69,70]. Amyloid A contains truncated TTR devoid of the *N*-terminal fragment, starting at approximately amino acid 49 [66,67,69,70,71]. Amyloid depositions of various morphologies, which were observed only in FAP patients, are small and contain long fibrils formed from mutated full-length TTR. The fibrils, known as amyloid B, are organized in parallel bundles [64,67]. Electron microscopy revealed amyloid fibrils in association with the basement membrane surrounding microvessels, which colocalized with amorphous deposits of nonfibrillar TTR forms observed in nerve biopsy [64]. Extensive studies on TTR pathophysiology, stability, and aggregation mechanisms resulted in two important therapeutic approaches/treatments against amyloidosis; one approach is directed to the clearance of TTR amyloid burden (siRNA-based, i.e., patisiran and anti-sense oligonucleotide-based therapies, i.e., Tegsedi^®^), and another approach is focused on TTR stabilization (tafamidis) [21,37,66].

## 3. Unaggregated TTR Is a Redox-Sensing Factor

TTR stability depends on the redox state of the environment, and TTR amino acid residues (particularly Cys10) are modified with different outcomes/effects by reducing and oxidative agents [55,56,72,73]. The free thiol of Cys10 of TTR in the plasma is most susceptible to oxidation, although there are many proteins more abundant than TTR in the plasma [74]. Consequently, amyloid formation propensity of TTR depends on redox conditions of the environment, and TTR was proposed to be a redox-sensing factor [75].

How TTR copes with oxidative stress is not fully understood; however, multiple connections of TTR with neuronal and pathological processes caused by oxidative and nitrosative stresses have been investigated [72,75]. In the brain, TTR helps to overcome the toxicity of the oligomers of misfolded proteins through multiple mechanisms, including TTR proteolytic activity [11,12,21,36,75]. TTR, especially in monomeric form, inhibits intracellular Ca^2+^ influx, reactive oxygen species (ROS) production, membrane permeabilization, and apoptosis, protecting SH-SY5Y cells and neurons against the stress induced by soluble oligomers of Aβ_42_ and the amyloidogenic *N*-terminal domain of the HypF protein from *Escherichia coli* [36]. Interestingly, TTR (mostly in a monomeric form) promotes the assembly of oligomeric proteins into larger and less toxic aggregates [36,76].

TTR helps TFAM to overcome the vicious cycle 1 (Figure 1A) of oxidative stress in AD [12]. TTR expression was strongly upregulated in the cortex (and in the hippocampus) of the brain in a mouse model of AD with hemizygous hTFAM transgene (ADh/hTFAMh). TTR upregulation helped TFAM to cope with stress induced by deposits of Aβ [12]. Interestingly, TTR colocalizes with TFAM in the mitochondria of TFAM-positive cells, and the formation of the TTR and TFAM complex in mitochondria was documented [12]. Sumoylation plays an important role in many crucial cellular functions, including stress response [77,78]. A transient increase in global sumoylation was observed in neuronal cells and astrocytes in a mouse model of AD [79]. The effect of Aβ on SUMO-1 conjugation was counteracted in neurons and astrocytes by cytotoxic stimuli, such as glutamate and oxidative stress [79]. These data indicate that pathological changes in global sumoylation caused by Aβ and oxidative stress may contribute to the pathogenesis of AD. Overexpression of TTR with sole SUMO-conjugating enzyme Ubc9 increases global protein sumoylation and may play a regulatory role in sumoylation by modulating cross-sumoylation of lysine residues of Ubc9 [80]. Cross-sumoylation of Ubc9 regulates the substrate specificity of Ubc9 and, thus, modulates cellular response to environmental conditions [80].

Importantly, TTR manifests its anti-oxidant properties under abnormal oxygen levels. TTR and certain plasma proteins, including hemopexin, alpha-1-antitrypsin, haptoglobin β-chain, apolipoprotein A1, and hemoglobin beta chain, were identified as candidates that can play a crucial role in adaptation to hypoxic stress due to limited oxygen availability [81]. In hyperbaric hyperoxia during saturation diving, TTR (and α-1-acid glycoprotein 1) is upregulated and oxidized (at methionine residues) [82]. The physiological response in hyperoxia includes physiological and biochemical changes to the immune system and an increase in the levels of ROS, heat shock proteins, nitric oxide, and cytokines [82].

In contrast to the anti-oxidant properties of TTR, the aggregated TTR forms induce oxidative stress [83,84,85,86]. The DJ-1 protease is an important regulator of hypoxia-induced response [87]. Under normal conditions, DJ-1 degrades TTR monomers, reducing amyloid formation [88]. Oxidative stress leads to abrogation of DJ-1 secretion from 293T cells, allowing accumulation of TTR amyloid. High levels of the inactive unoxidized form of DJ-1 were detected in FAP patients [88].

## 4. TTR and ECM Remodeling

TTR aggregation results in the disruption of extracellular matrix (ECM) homeostasis and changes the levels of matrix metalloproteinases (MMP), including membrane type MMP-14 and gelatinases MMP-2 and MMP-9, and tissue inhibitors in patients with FAP and cardiac amyloidosis (CA) [85,89,90]. Interestingly, in CA derived from ATTRwt amyloid, the circulating levels of MMP-2, MMP-9, tissue inhibitor of metalloproteinase-1 (TIMP-1), and mortality were lower than those in AL-derived CA [85,91]. MMP-2 and MMP-9 play a major role in ECM structure and remodeling, and in inflammatory response in the vascular tissue [92]. MMP-2 and MMP-9 may also contribute to fibrous cap thinning and plaque rupture in atherosclerosis [93], indicating a possible role of TTR in cap stability. Upregulation of ECM remodeling genes is indicative of imbalanced inflammatory processes that lead to pathological alterations in tissue structure and function [89,92].

Overexpression of TTR was shown to lead to upregulation of MMP-2 and MMP-9 in JEG-3 cells, increasing the migration and invasion of these cells [94]. JEG-3 cells are used as a model of trophoblast migration; hence, the data imply that TTR positively contributes to ECM remodeling during trophoblast migration and development. Disturbances in trophoblast migration and formation of the proper trophoblast vasculature lead to hypoxia and ischemia [94]. In preeclampsia, a complex and severe disorder of pregnancy, incorrect placental development was shown to be correlated with TTR levels, spatial distribution, and stability, and low levels of TTR were detected in the plasma and syncytiotrophoblasts [33,95]. Importantly, TTR aggregation is responsible for preeclampsia development, and unaggregated TTR prevents the onset of a pathological phenotype [33,96]. Therefore, in addition to hormonal transport, the contribution of TTR to placental development involves the regulation of ECM remodeling.

In amyloid-related diseases, universal amyloid-associated proteins (including apolipoprotein E, serum amyloid P component, proteoglycans, such as heparin sulfate and perlecan, and proteins remodeling ECM) colocalize with disease-causing amyloid protein [89]. TTR was shown to bind perlecan, which is the main constituent of the basement membrane [27]. Biglycan is involved in the binding and organization of collagen fibrils and was shown to colocalize with TTR fibrils [89]. These results suggest that TTR interacts with or is functionally connected to the major constituents and enzymes responsible for ECM structure and remodeling and inflammatory response in the vascular tissue. Interestingly, sulfated glycosaminoglycans, which are anionic polymers present in the ECM (especially heparin), were shown to accelerate aggregation of TTR (and other amyloid-forming proteins) by promoting conversion of soluble oligomers into less toxic insoluble amyloid fibrils [97]. Anionic phospholipids in the lipid membranes also promote aggregation of mutant and wild-type TTR, and the binding is proportional to TTR aggregation [98,99].

## 5. Link between TTR and Ca^2+^

Despite extensive studies, detailed molecular mechanism of TTR amyloid formation are incompletely understood. In particular, the investigation of the role of physiological factors in the regulation of TTR stability should be continued. Amyloid formation can be associated with metal-induced oxidation (Zn^2+^, Cu^2+^, Fe^3+^, Mn^2+^, Co^2+^, and Ca^2+^) and subsequent *N*-terminal backbone fragmentation of TTR molecules [47,100]. Metal-induced cleavage of the N-terminus (mostly at the Cys10-Pro11 peptide bond) results in simultaneous appearance of new conformers and increases the propensity of TTR to aggregate [47,100]. Particular attention should be directed toward the role of Ca^2+^ in the stability of TTR structure and function because Ca^2+^ regulates many essential physiological processes of the cells, tissues, and organs.

Investigation of neurotrophic and neuroprotective roles of TTR revealed that, in neurons of the central nervous system, unaggregated wild-type TTR is bound to the megalin receptor (LRP-2) and is internalized via endocytosis by neuronal cells [29,101]. Binding of TTR to megalin induces Src-regulated signaling pathways, leading to transient increase of intracellular Ca^2+^ concentration via N-methyl-d-aspartate receptors and activation of CREB via MAPK pathway [29]. Importantly, TTR deposits, especially HMW oligomers, induce the influx of Ca^2+^ from the extracellular milieu into the cells [83,102,103]. Induced stress response causes intracellular Ca^2+^ efflux from the ER, leading to caspase 3 activation [84]. TTR oligomers were also shown to be internalized into the cells (i.e., cardiomyocytes) [83]. On the other hand, high concentrations of Ca^2+^ induce destabilization of TTR tetramers, contributing to TTR oligomerization and amyloid formation [47]. Therefore, there is a bidirectional relationship between TTR stability (and, at least indirectly, TTR function) and intra- and extracellular Ca^2+^ pools.

Early observations on the binding of metal cations by TTR [104,105] were confirmed and extensively studied in the case of Zn^2+^, Cu^2+^, and Fe^3+^ [54]. However, the data on the interactions between Ca^2+^ and TTR are scarce [47]. Two crystal structures of TTR complexed with Ca^2+^ are deposited in the PDB database (https://www.rcsb.org/, accessed on 21 February 2019, 4n85 and 4MRB), and Ca^2+^ binding does not impose significant conformational change on the TTR structure [47]. However, Ca^2+^ was shown to affect conformational flexibility, which leads to lower thermal stability of the TTR tetramer. Thermal unfolding of TTR in the presence of Ca^2+^ resulted in lowering (by 3 °C) of the melting temperature (T_m_) [47]. These observations indirectly indicate that unfolded non-native form of TTR may have a certain affinity for Ca^2+^. Lower stability of TTR in the presence of Ca^2+^ may lead to TTR aggregation in the presence of sufficiently high local Ca^2+^ concentration. Subsequent Ca^2+^ binding by aggregated TTR should result in slow accumulation of Ca^2+^ depositions. Likewise, microcalcifications are commonly observed in ATTR cardiac amyloidosis (ATTR CA) [106]. Interrelation of TTR stability and Ca^2+^ creates vicious cycle 2 (Figure 1B); TTR amyloid is formed in situ in response to high Ca^2+^ concentrations, which in turn leads to further TTR destabilization and promotion of amyloid deposition, leading to the accumulation of Ca^2+^ deposits.

## 6. Involvement of TTR in Biomineralization

Proteomic analysis identified TTR as one of the proteins specifically associated with mammillary cones and important for the initial phase of eggshell biomineralization in chicken [107]. Investigation of the ability of TTR to influence the formation and morphology of calcium carbonate crystals demonstrated that extensive and amorphous deposits of human TTR are associated with crystals with unique morphology [108]. Additionally, various amyloid-like TTR forms, which remodel crystal faces, were present on the inner and outer surfaces of the crystals [108]. Morphology of large crystals is strikingly similar to that of the crystals obtained in the presence of MRCP20 protein, which is involved in biomineralization of the cement of barnacle *Megabalanus rosa* [108,109,110]. MRCP20 initiates the formation of amyloid-like fibrils on the surface of mineral crystals through stable β-sheet motives [111]. Some calcium carbonate crystals grown in the presence of TTR were small and rod-shaped [108]. Rod-shaped structures were also observed in the barnacle cement [112], indicating functional similarity between MRCP20 and TTR. Currently available information about the nucleation stage of biomineralization indicates the importance of the amyloid and disordered protein components in the formation of the polymer-induced liquid phase (PILP) in the presence of ions [113,114]. Thus, it was postulated that TTR amyloid may play a functional role in biomineralization, e.g., in the cement line, which constitutes the mineralization front between the subchondral bone and calcified cartilage [108,115].

## 7. Calcium-Containing Protein–Mineral Nanoparticles

Rod-shaped crystals obtained in the presence of TTR in in vitro biomineralization assays resemble mineral structures called nanobacteria, which were detected in body fluids [108,116,117]. Despite previous assumptions of biotic origin, nanobacteria belong to the group of protein–mineral nanoparticles (calcifying nanoparticles, CNPs) [116,117,118]. CNPs were isolated from human blood and were shown to be associated with numerous pathological vascular calcifications and calcification-related diseases, such as atherosclerosis, heart valve calcification, stone formation, renal tubular calcification, and even AD [119,120]. The presence of CNPs induces an inflammatory response in the tissue [119,120]. Recent data indicate that protein–mineral nanoparticles have heterogeneous nature, origin, and function. In addition to nanobacteria/CNPs, protein–mineral nanoparticles also include calciprotein particles and other protein–mineral complexes [117,118,120]. Calciprotein particles are formed in body fluids and are dynamic/reorganizing colloidal protein–mineral complexes composed of acidic proteins and calcium phosphate [118,121]. These particles maintain the liquid mineral phases at local supersaturation during biomineral formation and help to solubilize excess biominerals for elimination, preventing mineral deposition in the extracellular space [118]. Calciprotein particles isolated from the blood of uremic patients contain mostly fetuin A, albumin, apolipoproteins, and components of the complement system [118,121]. Protein components of protein–mineral nanoparticles indicate the close relationship of blood proteins involved in wound healing, metabolism, and the immune system with biomineralization. TTR is one of the proteins associated with the protein–mineral nanoparticles formed in the presence of human serum, CSF, and pleural effusion groups [122], and with hydroxyethyl starch-coated magnetite nanoparticles formed ex vivo [123].

Interestingly, studies on the formation of calciprotein particles containing fetuin A demonstrated that mineral clusters are stabilized by the β-sheet-rich cystatin-like domain of fetuin A (CY1) [118]. The mineral-binding domain CY1 is covered by an intrinsically disordered *C*-terminal region that has to be displaced to enable interaction of calcium phosphate with CY1. This interaction underlies the formation of the amorphous (colloidal) protein–mineral hydrated phase, which is a precursor of biomineral deposits [113,118]. The connection between mineralization and protein aggregation results from the formation of the mineral phase through amorphous protein–mineral phases, such as PILP, that undergo subsequent stepwise dehydration [113,118]. Protein flexibility (due to unstructured regions and/or amyloidogenic properties) is important for PILP formation [113].

## 8. TTR Involvement in Noncardiac Disorders

In agreement with the in vitro data on the involvement of TTR in mineral formation, several lines of evidence suggest that TTR is connected to mineralization and/or calcification in vivo. First, TTR was shown to bind to hydroxyapatite in vitro or in vivo at the tooth enamel surface [124,125]. Moreover, extensive TTR depositions were observed in the majority of normal aged human cartilage [126,127] and ligaments [127,128]. Additionally, abnormal TTR levels were observed in the diseases of cartilage and bone tissues (Table 1). TTR downregulation has been detected in the plasma of rheumatoid arthritis (RA) patients [129]. The proteomic approach and statistical analysis allowed for the creation of the pathway maps, process networks, and GO processes that were associated with RA. Interestingly, the most statistically significant (the lowest *p*-values) data were obtained for reverse cholesterol transport (pathway maps), blood coagulation (process networks), single-organism transport, and innate immunity (GO processes). TTR, gelsolin, angiotensinogen, lipopolysaccharide-binding protein, and protein S100-A9 were selected as the set of biomarkers to discriminate healthy persons from RA patients [129]. Four different forms of TTR were detected in mass spectra of sera from patients with RA, early RA, osteoarthritis (OA), and healthy control group [130]. These forms corresponded to unmodified and post-translationally modified (at amino acid residue Cys10) TTR molecules with sulfate (Sul-TTR), cysteine (Cys-TTR), and cysteinylglycine (Cysgly-TTR). The proportions of different forms of TTR were unique for each patient group. In the early RA patient group, TTR level was higher than in the late RA patient group or in the healthy control group. There was also an increase in Sul-TTR and decrease in Cys-TTR levels compared to the healthy control group. Thus, it was concluded that TTR may be a serological marker for early RA [130]. RA is a chronic autoimmune disorder that leads to inflammation of the cartilage and bone destruction, and the innate immune system contributes to pathogenesis of the disease [131]. Upregulation and high levels of aggregated and oxidized forms of TTR were detected in the plasma and synovial fluid in juvenile idiopathic arthritis (JIA) [132]. HMW complexes ranging from 240 kDa to 1000 kDa, formed via noncovalent interactions between TTR molecules, were observed in synovial fluid of JIA patients. Inflammation was postulated to induce ROS, which are responsible for TTR oxidation and drive the aggregation of TTR. Oxidized and aggregated TTR induces the immune response and production of autoimmune anti-TTR antibodies [132].

TTR deposits were also detected in the cartilage of patients with OA [126] and were linked to inflammation and disease progression [126,134]. The injections of aggregated TTR promoted the expression of catabolic and inflammatory mediators in the joint tissue [134]. TTR molecules in the plasma are *N*-terminally truncated, and the plasma levels of truncated TTR form are reduced in OA patients with active disease [133]. Calcification of the articular cartilage is an active process and contributes to cartilage degeneration and damage in OA [137,138]. Although OA is considered a disease of the cartilage, and the destruction of articular cartilage is an indicator of OA severity [137], recent evidence suggests the presence of intercellular communications between subchondral bone and cartilage in OA [139,140]. Interestingly, hypoxia and differential expression of mediators of the vascular system in subchondral bone were shown to underlie OA pathogenesis [139]. These observations demonstrated the contribution of TTR aggregation, calcification, and the immune and vascular systems to pathophysiology of OA. Unexpectedly, deletion of the TTR gene was shown to increase the severity of OA [134], indicating that TTR may play an unknown role in OA development and/or that loss of TTR function contributes to OA pathogenesis.

In women with osteoporosis and in older adults with type 2 diabetes mellitus (T2DM), the plasma level of TTR was specifically associated with low bone mineral density [135,136]. Inverse correlation between the levels of TTR and inflammation markers (hs-CRP) suggests a protective role of TTR in osteoporosis [135]. However, TTR knockout in mice resulted in increased bone mineral density and trabecular volume and elevated peptidylglycine α-amidating mono-oxygenase (PAM) expression in the brain and bone [141,142]. NPY amidation by PAM is required for NPY activity in bone homeostasis; therefore, a study concluded that TTR affects bone mineral density by negative regulation of PAM and NPY amidation [142]. However, the activity of PAM may regulate other proteins involved in mineralization and bone cell physiology and development; therefore, the effect of TTR on the bone tissue may be complex [143]. Interestingly, PAM is linked to T2DM and heart disease [144]. A recent report showed that the presence of PAM (but not PAM mono-oxygenase activity) is required for the formation of secretory granules of atrial natriuretic peptide precursor in cardiomyocytes [145].

Overall, the data on the engagement of TTR in osteoarticular disorders show that TTR participates in the health of bone and articular tissues, particularly in the immune and calcification processes. Whether these TTR roles are physiological or pathological, and which role is played by the aggregated form and which role is played by the unaggregated form of TTR remain open questions. The involvement of TTR in osteoarticular disorders is an indirect indication of TTR involvement in calcification occurring in cardiovascular disease. Osteoarticular/ligament disorders precede or coincide with ATTR CA, which is associated with aortic and/or valve stenosis caused by calcification [127,146,147].

## 9. TTR Contribution to Cardiovascular Disease and Vascular Calcification

Coronary artery disease (CAD) is an inflammation-related chronic disorder caused by blockage of coronary arteries by atherosclerotic plaques. CAD (in addition to hypertension) is a major risk factor for heart failure (HF) [148]. Two-dimensional gel electrophoresis and MALDI-TOF MS/MS analyses identified TTR among proteins differently expressed in CAD patients [149]. Because a low level of TTR in the plasma of CAD patients (observed also by Western, ELISA, FACS, and by in silico analysis) may be correlated with disease severity, TTR has been proposed as a marker for CAD screening, with an indication that further large-scale studies are needed [149]. Low TTR levels are generally observed in cardiovascular disorders, particularly disorders associated with T2DM and/or calcification (Table 2). Low TTR levels are positively correlated with coronary artery stenosis (caused by valve calcification) and with angiographic severity of acute coronary syndrome (ACS) [150]. An increase in the oxidized form of TTR was observed and is correlated with arterial stiffness and cardiovascular risk in T2DM patients [151]. Additionally, in ATTR CA, a decrease in the plasma concentration or stability of TTR is associated with shorter survival [147,152]. Low molecular weight stabilizers (tafamidis, AG-10) bind TTR and reduce mortality, restore plasma levels, and/or improve the outcome of ATTR CA patients [147,153]. These observations suggest that even partial deprivation of functional TTR contributes to calcification and to occurrence/severity of cardiovascular disease (CVD). In ATTR CA, a low level of TTR can be linked to TTR aggregation.

In CA, misfolded and aggregated forms of proteins are cytotoxic and underlie the pathogenesis of the disease [159]. Mutations, improper post-translational modifications, aging factors, and altered proteostasis contribute to destabilization of the structure of amyloidogenic proteins [159]. Some TTR mutations result in the early onset of ATTR cardiomyopathy and polyneuropathy with variable outcomes [147,160]. Cytotoxicity of TTR amyloid results in the induction of oxidative stress and modification of mitochondrial potential, which lead to cardiac dysfunction [83]. Concomitantly, ATTR CA is strongly associated with HF [160,161]. Amyloid deposits of wild-type TTR fibrils are observed in patients with heart failure with preserved ejection fraction (HFpEF), and TTR amyloid infiltration is a pathological factor in HFpEF clinical syndrome [156,162]. Pathogenesis of HFpEF involves cardiomyocyte stiffness due to abnormal Ca^2+^ and disturbances in ECM homeostasis associated with inflammation [163]. Both oligomers and fibrils of TTR were shown to interact with the plasma membrane of cardiomyocytes [83]. Cytotoxicity of the misfolded forms of TTR to cardiomyocytes involves ROS generation and disturbance of mitochondrial potential and cytoplasmic Ca^2+^ homeostasis [83]. TTR aggregates and fibrils dysregulate cytoplasmic Ca^2+^ balance and cycling in exposed excitable cells. TTR oligomers, but not TTR fibrils, are internalized [83]. Interestingly, in an ex vivo experimental system using HL-1 cardiomyocytes, the amyloid/fibrillar forms of TTR, induced effects that were milder and had different response times than those induced by natively folded mutated and nonmutated TTR [164]. An increase in ROS production induced by tetrameric and aggregated forms of TTR was similar. These effects were associated with changes in autophagy, lipid membrane fluidity, and metabolic and phosphorylation processes, indicating that various forms of TTR differ in their interaction with the membranes and exert variable pathological (or physiological) effects, particularly on the mechanisms of Ca^2+^ release/imbalance [164].

The amyloid deposits of misfolded TTR are detected/imaged using bone scintigraphy with technetium-99 probes (bone tracers), which are fairly specific for ATTR CA [106,165,166]. The specificity of bone tracers for ATTR CA may be due to increased levels of microcalcification detected in TTR depositions compared to the deposits formed by immunoglobulin light chain amyloid (AL) [167]. Additionally, ATTR CA is associated with aortic stenosis caused by calcification [146]. CVD is interconnected with vascular calcification (VC), which is a highly regulated and cell-mediated phenomenon resembling the process of physiological ossification in many aspects [168]. Changes in the phenotype and viability of vascular smooth muscle cells (VSMCs) under stressed conditions are crucial for VC [168,169]. Mitochondrial and ER stress and defective autophagy contribute to phenotypic switching of VSMCs. Several processes link bone turnover with soft tissue calcification, and some data indicate that impaired bone turnover promotes VC [170]. VC is not a simple consequence of high calcium and phosphorus conditions/environment, and also represents an imbalance in anti-calcific and osteochondrogenic signals. Dysregulation of Ca^2+^ homeostasis underlies VC [168].

Calcific aortic valve disease (CAVD) is a slow and progressive disorder of calcification occurring in the aortic valve. Calcification in CAVD ranges from mild aortic sclerosis without blood flow obstruction to severe calcification that causes valve thickening and stenosis [171]. The molecular mechanisms of CAVD are similar to those characteristic for pathogenesis of atherosclerosis [171]. Complex analysis of the transcriptome, proteome, and miRNA datasets differentially expressed in CAVD allowed the creation of a three-dimensional multilayered model of the disease [155]. The 3D network of interactions of seven input genes, four input miRNAs, and two connectors indicated that two amyloid-forming proteins, amyloid β precursor protein (APP) and TTR, play central roles in the processes underlying CAVD [155]. Therefore, the network indicated a connection between cardiovascular and amyloid diseases. Amyloid depositions of APP were localized to the fibrocalcific region of the aortic valve using immunohistochemistry, and TTR deposits were detected in both calcified valves and the fibrocalcific region of the aortic valve in CAVD patients [155]. Analysis revealed the importance of the crosstalk of the coagulation/complement cascade pathway with platelets in etiology of the disease [155]. Increased risk of thromboembolism observed in CA [172] is in good agreement with these observations. Thus, associations between amyloid formation, calcification, and the coagulation/complement system with crucial involvement of TTR were detected in CAVD. Therefore, TTR aggregation may be implicated in calcification of the aortic valve in CAVD.

In CVD, in hemodialysis patients with T2DM, low TTR plasma levels are associated with increased mortality [154]. Diabetes mellitus is a metabolic disorder of chronic hyperglycemia that increases the risk of atherosclerosis, microvascular disease (including retinopathy, nephropathy, and neuropathy), and macrovascular disease, and ultimately leads to VC [173,174,175,176]. T2DM patients have increased coronary artery calcification scores, calcified plaque burden, and risk of developing tibial artery calcification [173]. In T2DM progression, ROS, dysregulation of mitochondria and miRNA, and inflammation play various pathological roles depending on the lifestyle and genetic factors [174]. Cysteinylated TTR, which is an indicator of oxidative stress, is positively correlated with cardiovascular risk and vascular stiffness in T2DM patients [151]. In T2DM, improper hormonal signaling caused by dysfunction of β- and α-cells of the pancreas results in insulin insufficiency and disturbances in lipid metabolism [174]. TTR has been shown to contribute to the functioning of pancreatic β- and α-cells [7,8]. TTR is important for glucagon release from α-cells [8], contributing to glucose homeostasis. Notably, tetrameric TTR protects β-cells against apoptosis and stimulates insulin release, coupled to an increase in the intracellular Ca^2+^ concentration [7]. TTR binds to glucose-regulated protein Grp78 and is internalized into pancreatic β-cells [177]. 

GRP78/BiP is a master regulator of ER homeostasis that activates adaptive stress-induced response (unfolded protein response, UPR) [178]. The accumulation of misfolded proteins (including TTR) induces UPR via GRP78/BiP chaperone [84,102]. Prolonged ER stress leads to apoptosis [178,179]. ER stress is linked to ROS and inflammation, and is crucial for the development and progression of heart-related diseases, including CVD and ATTR CA [84,159,173,174,180,181]. Protein aggregates also result in remodeling of the ECM, a process that is intimately connected to vascular disease [92]. Therefore, in the following sections, the contribution of TTR to the pathogenesis of atherosclerosis and balance between fibrinolytic and coagulation systems are considered.

## 10. TTR and Wound Healing (Fibrinolysis vs. Thrombosis)

Adsorption of the proteins on the surfaces drives the structural changes in adsorbed proteins [182,183]. Contact of the blood with negatively charged surfaces (glass) or polymers, such as polyphosphates, some nucleic acids, glycosaminoglycans, or aggregated proteins, induces activation of the coagulation cascade by the contact system of the blood [184]. Aggregated proteins containing cross-β structures (but not native or fibrillar structures) have been shown to activate fibrinolytic and contact systems of blood coagulation, inducing an inflammatory response and degradation of fibrin polymers [184]. Therefore, TTR amyloid may function in the activation of coagulation and fibrinolytic systems and/or contribute to the regulatory network of coagulation and fibrinolytic balance.

Plasmin plays a crucial role in fibrinolysis, wound healing, cell signaling, inflammatory regulation, and atherogenesis [185,186,187,188,189]. Plasmin is secreted as a zymogen (plasminogen) that is activated by proteolytic cleavage by tissue-type plasminogen activator (tPA) or urokinase-type plasminogen activator. Plasmin activity is an important component of the complex regulatory network balancing coagulation (fibrin clot formation) and fibrinolysis (fibrin clot degradation). Perturbation in coagulation and fibrinolytic homeostasis can lead to bleeding disorders or thrombus formation [187]. Plasmin activity is controlled by inhibitors of plasmin activators or directly by α2-antiplasmin [185]. Plasmin induces apoptosis of VSMCs, thus destabilizing atherosclerotic plaques and increasing the possibility of rupture events [190]. Recently, plasmin was shown to specifically cleave TTR, which results in the formation of TTR fibrils containing truncated TTR molecules (49–127) [190]. Plasmin-derived TTR fibrils resemble the fibrils isolated from TTR amyloid formed in vivo [70]. These observations indicate that physiological fibrinolysis in vivo is interconnected with the formation of TTR amyloid [190]. Since plasmin activation occurs in vivo at sites of injury (i.e., on vascular and arterial walls or vascular basement membrane), an intersection between TTR amyloid formation and the fibrinolytic system corresponds to previous observations concerning CAVD [155]. Additionally, cleavage-induced formation of TTR amyloid may lead to local microcalcification in situ due to the binding of Ca^2+^ by destabilized TTR molecules [47,108]. Multiple routes of injury and healing processes can cause accumulation of microcalcifications in the vascular tissue. This mechanism correlates with slow and progressive calcification observed in CAVD [171] and with selective staining of TTR amyloid deposits by bone tracers in cardiovascular calcification [165,166].

The domination of fibrinolytic activity over coagulation activity results in a risk of hemorrhage observed in diseases caused by TTR or other amyloid-forming proteins [191,192,193]. Patients with SSA caused by an amyloid other than ATTRwt had elevated plasmin-α2-antiplasmin levels, resulting in elevated plasmin generation and high fibrinolytic activity [193]. Permanently elevated plasmin-α2-antiplasmin levels result in constitutive activation of the fibrinolytic system, which explains the bleeding tendency observed in SSA [193]. The authors suggested that the fibrinolytic system in SSA patients is activated directly by misfolded and aggregated proteins [193]. The lack of elevation of plasmin-α2-antiplasmin levels in SSA caused by TTR aggregation indicates that TTR plays a more complex role in the fibrinolytic/coagulation system. TTR is both a substrate for proteolysis and a protease. Does TTR play a role as a protease in the coagulation cascade?

Extracellular amorphous protein aggregates (but not fibrils or aggregates containing cross-β-structures) bind plasmin, tPA, and plasminogen via an exposed lysine-dependent mechanism [194]. This binding protects plasmin from α2-antiplasmin inhibition and contributes to plasmin activation. Plasmin digests amyloid, and the fragments are then bound by chaperones and removed by lysosomes in endothelial and microglial cells [194]. Accordingly, in the plasma of FAP patients, extracellular chaperones are overrepresented, and increased proteolytic activity is observed [195]. However, amyloid-β was shown to be bound by fibrinogen to form protease-resistant amyloid-fibrin clot that shields Aβ from plasmin degradation [196]. Therefore, fibrin-bound amyloid delays the proteolysis of amyloid by plasmin, creating a regulatory loop ensuring a fine balance between activation of plasmin by amyloid and amyloid degradation by plasmin. Consequently, TTR, together with other proteins (α-1-antitrypsin, α-1-antichymotrypsin, α-1-acid glycoprotein, inter-α-trypsin-inhibitor, complement proteins, haptoglobin, and fetuin-A), was detected in platelet-rich fibrin [197]. Platelet-rich fibrin stimulates osteoblastic differentiation and proliferation of human bone mesenchymal cells, and increases regeneration of the bone and fibroblast activity in the ligament [197]. Formation of functional TTR amyloid may occur in blood clotting and mineralization processes. The evolutionary link between calcification and amyloid formation and blood coagulation was also postulated for cement polymerization in barnacle *Megabalanus rosa* [198]. The *Megabalanus rosa* cement proteins MRCP20 and TTR are involved in the formation of protein–mineral phases that link amyloid formation with biomineralization. 

The involvement of TTR in fibrinolysis–coagulation balance confirms the connection of TTR level with venous thromboembolism [157]. TTR was shown to be the strongest plasma biomarker candidate for future venous thromboembolism [157]. Imbalance between coagulation and fibrinolytic systems (emphasized by local hypoxia) is the major cause of thromboembolism [199]. Hypoxia promotes pro-coagulative conditions [199]. Considering that TTR is upregulated under the hypoxic conditions [81], TTR upregulation may represent a protective response to thromboembolism. Interestingly, DJ-1 is another candidate biomarker with a stronger effect, and an inverse correlation between plasma levels of TTR and DJ-1 (which is a protease digesting TTR) has been detected [157]. In addition to TTR and DJ-1, other plasma proteins (mostly involved in the complement and coagulation systems) were associated with thromboembolism [157].

Infection with SARS-CoV-2 results in a disturbance of fibrinolytic homeostasis, leading to a hypercoagulable state and thrombotic complications [200]. Plasma levels of TTR are directly correlated with pro-coagulation activity in the blood [157]. Recently, the plasma concentration of TTR was shown to be positively associated with the outcome of COVID-19 [201]. “Thrombosis supports innate immunity” because the mechanisms of initiation and propagation of deep vein thrombosis are evolutionarily linked to immune defense induced by pathogen infection [202]. The dominance of pro-coagulative activity has at least two functional consequences, leading to a decrease in TTR cleavage by plasmin and to abundant extravascular fibrin depositions that are observed in the pathogenesis of lung injury [203,204]. Associations of TTR with severity of COVID-19 confirm that TTR contributes to a balance between fibrinolytic and coagulation systems.

## 11. TTR Interconnection with Inflammation

The deposition of aggregated proteins (including fibrin) activates tPA and factor XII [184]. tPA activation directly leads to an increase in plasmin levels (an increase in fibrinolytic activity). On the other hand, the activation of factor XII leads to the activation of the intrinsic coagulation pathway and results in the formation of bradykinin, a vasoactive peptide that promotes/induces multiple inflammatory responses [184].

On the other hand, properly folded TTR is a negative marker of inflammation and a positive marker of nutrition status, guarding the homeostasis of protein synthesis and breakdown [32,40]. However, elevated ROS, oxidative modifications, and aging negatively affect the structural stability of proteins in general, and TTR in particular, and lead to TTR aggregation [55,56,72,205]. In turn, TTR amyloid induces a cytotoxic response that involves ER stress, dysregulation of Ca^2+^ balance, induction of UPR, upregulation of MMPs, apoptosis, and binding of membrane lipids [85,90,91,100,206]. Consequently, TTR amyloid contributes to oxidative stress and progression of inflammation [86,89,207,208]. In the plasma of FAP patients, the levels of cytokines (such as TNF-α, IL-1β, IL-8, IL-33, IFN-β, IL-10, and IL-12) are altered compared to those in healthy individuals [208]. Changes in cytokine levels were also observed in asymptomatic FAP patients, indicating that the induction of inflammation precedes amyloid fibril deposition [208].

Stabilization of proper TTR structure reduces cytotoxicity induced by TTR aggregates and improves survival of patients with ATTR CA [66,153]. However, the factors that reduce oxidative stress, such as tauroursodeoxycholic acid (TUDCA), were also shown to lead to a reduction in toxic aggregates of TTR [207]. TUDCA does not affect the stability of TTR in vitro, suggesting that the association of TTR with oxidative stress and inflammation is indirect and bidirectional, and that oxidative stress leads to TTR destabilization and amyloid formation [207]. This bidirectional relationship between amyloid and inflammation also occurs in AD. Aβ peptide deposits induce immune response, and molecules involved in inflammatory processes can increase the formation of Aβ [209,210]. Interestingly, inflammatory processes that lead to Aβ deposition are induced by peripheral plasmin [209]. Chronic inflammation leads to amyloidosis, and amyloid activates immune signaling [159,209,210]. Since inflammation, in turn, accelerates protein aggregation, this interconnection drives vicious cycle 3 (Figure 1B).

In blood exposed to inflammatory concentrations of hypochlorous acid, the oxidation of amino acid residues and dityrosine crosslinking of TTR with other plasma proteins (such as α1-antitrypsin, haptoglobin, and albumin) was detected [205]. HMW aggregates of oxidized proteins also include α, β, and γ fibrinogen chains, apolipoprotein A1, and complement C3. In vivo, hypochlorous acid is produced by myeloperoxidase (MPO), which is present in mammalian neutrophils, monocytes, and some subtypes of tissue macrophages [206,211]. MPO is the key enzyme in the host innate immune defense against pathogen infection [206,211]. Elevated levels of MPO activity in the plasma are observed in myocardial infarction and in ACS, and high MPO concentrations are associated with major adverse cardiovascular events in patients with CAD [206,212,213]. The yin and yang action/dual nature of MPO is manifested by the destruction of invading pathogens and MPO involvement in disease progression and harmful effects on the host tissues [206].

## 12. TTR Role in Atherosclerosis

Atherosclerosis is a chronic inflammatory disease linked to oxidative and ER stresses [214]. In the early stages of atherogenesis, the enzymes involved in nonoxidative LDL modification (including plasmin, MMT-2, and MMT-9) are activated, initiating the cascade of reactions promoting the development of atherosclerosis [186,215]. Oxidative modifications of lipoprotein-B-containing lipoproteins (LDL and Lp(a)) within the vessel walls occur at early stages of atherogenesis [216,217]. Lp(a) is a form of LDL modified by covalent binding with apolipoprotein(a), whose structure is similar to plasminogen [218]. Due to this similarity, Lp(a) forms a complex with fibrin, plasminogen, and tPA, resulting in a decrease in fibrinolysis [217,218]. Consequently, Lp(a) is a prothrombotic molecule with many proatherogenic and pro-inflammatory effects [217,218]. VSMCs protect the vessels from proteolytic injury caused by atherosclerosis by expressing high levels of anti-plasmin proteins and endocytic LDL-receptor-related protein-1 (LRP-1), which is able to capture aggregated LDL and plasmin–antiplasmin complexes [215]. LRP-1 is critical for lipid metabolism and plays an essential, although complex (mainly protective), role in atherosclerosis [219]. LRP-1 is also expressed in other types of cells, including neurons [219]. TTR mediates the clearance of Aβ from the brain via binding and unidirectional transport of Aβ through LRP-1-mediated endocytosis [220]. Importantly, downregulation of LRP-1 was observed in TTR (-/-) mice, and in endothelial and hepatocyte cell lines incubated in the absence of TTR, indicating that TTR positively regulates the expression of LRP1 [220]. Therefore, TTR may also play a protective role via LRP-1 receptor in atherosclerosis, especially because LRP-1 is involved in glucose homeostasis and inflammation, and binds HDL [219,221].

The protective role of HDL, a natural nanoparticle, in atherosclerosis is generally accepted [221,222]. In addition to reverse cholesterol transport, HDL has anti-oxidant and anti-inflammatory properties and anti-thrombotic activity, and contributes to intercellular signaling [221]. In the plasma, TTR associates with ApoA1 and is thus present within circulating HDL, contributing to the protective functions of HDL [28,221]. Interestingly, an overlapping set of plasma proteins (TTR, ApoA1, α-1-antitrypsin, fibrinogen alpha chain, and C3 complement protein) was shown to be associated with both HDL and oxidative stress response induced by MPO [205,221]. This observation indicates that a particular set of molecules associated with HDL, in addition to the quantity of HDL or concentration of cholesterol-loaded HDL-C, determines the molecular functions of HDL [221], and that HDL is functionally related to the defense mechanisms of the innate immune system against pathogen infection.

Anti-thrombotic activity of HDL [223] corresponds to TTR involvement in the thrombotic phenotype [157]. Similarly, the transport of miR-223 by HDL in patients suffering from familial hypercholesterolemia [221,224] correlates with the role of TTR in the MEG3/STAT4/miR-223-3p/FBXW7/Notch1 signaling pathway (see next section) [225,226]. Anti-inflammatory (anti-atherogenic) effects of HDL were shown to be due to miR-223 delivery and downregulation of intercellular cell adhesion molecule 1 on endothelial cells that do not express miR-223 [224].

Recent information about HDL function indicates that HDL can be anti-inflammatory or pro-inflammatory particles, depending on environmental needs [221]. In acute viral infection and in chronic inflammatory diseases, such as atherosclerosis and RA, HDL enhances the inflammatory response [227]. Exchange of anti-inflammatory (ApoA1) for pro-inflammatory (serum amyloid A, SAA) components creates a functional switch [227]. TTR cleaves ApoA1, contributing to the formation of ApoA1 amyloid [228]. Amyloid depositions of *N*-terminal fragments of ApoA1 were detected in the tunica intima of arterial vessels and in atherosclerotic plaques [229]. ApoA1 proteolysis decreases the reverse cholesterol flux mediated by HDL [228]. However, the proteolysis of ApoA1 by TTR may be protective by facilitating ApoA1-SAA exchange and HDL transformation from anti- to pro-inflammatory functions. This observation is in agreement with a positive correlation of TTR levels with the outcome of SARS-Cov-2 infection [201]. Pro-inflammatory exchange of HDL protein subunits was observed in COVID-19 patients [230].

The thiol groups of the cysteine residues of TTR and other plasma proteins (MMT, fibronectin, albumin, and Lp(a)) were shown to form disulfide bridges with homocysteine (HCy) [231,232]. HCy is a pro-oxidant molecule that plays pro-inflammatory roles [216,231]. The thiol group of HCy is easily oxidized to induce ROS, which cause damage to the endothelium [216,233]. An elevated level of HCy in the blood is a risk factor for CAD, cerebrovascular disease, venous thromboembolism, osteoporosis, and AD [216,231]. HCy activates platelets and changes normal anti-coagulant activity of blood cells to pro-coagulant activity [231]. HCy increases Lp(a) binding to fibrin and delays fibrinolysis [217,231]. The ratio of HCy to unmodified TTR reflects the total HCy level in the plasma of patients with renal disease and is an indicator of plasma HCy overload [231,232]. Importantly, HCy, Lp(a), and fibrin are localized in atherosclerotic plaques [222,223,234], and TTR was also detected within the plaques in carotid arteries [235]. The distribution of TTR (and other proteins) in the plaques is sex-dependent, and TTR is upregulated in the plaque center (especially in fatty streak and shoulder) in women [236]. Women tend to have younger and more fibrous plaques than men, and the plaques in men are more atheromatous, associated with neovascularization, cellularity, and inflammatory infiltration [236,237,238]. Higher expression of TTR in the plaques in women suggests a functional role of TTR in sex-related differences in atherosclerosis [236].

Stability of the arterial plaque is negatively influenced by neovascularization and positively influenced by calcification, although micro- or irregular calcification may increase the risk of plaque rupture [233,239]. Recent studies have shown an inverse correlation between calcification of atherosclerotic lesions and probability of plaque rupture in CAD [234]. Paradoxically, extensive calcification, which represents the late advanced stage of atherosclerotic plaques, is more resistant to thrombotic events causing ACS. A greater extent of calcification relative to the plaque area characterizes healed stable plaques with lower local inflammation [234]. Calcification localizes close to lipid deposits, and groupings of calcified lipid microvesicles form large clumps of calcifications [233]. Notably, cholesterol and anionic phospholipids in lipid membranes bind TTR and can induce TTR aggregation [98]. On the other hand, aggregated TTR alters membrane fluidity and induces cytotoxic effects [99], creating vicious cycle 4 (Figure 1B). Therefore, lipids may contribute to amyloid formation and subsequent calcification of atherosclerotic plaques. Anti-angiogenic activity may be another functional role of TTR in carotid plaque.

## 13. TTR Regulation of Angiogenesis

Transcriptome analysis of human retinal endothelial cells (hRECs) in diabetic retinopathy (DR) revealed the regulatory network of protective functions of TTR, implying that TTR co-ordinates oxidative stress, inflammation signaling, autophagy, and apoptosis in DR [226]. DR is a serious microvascular complication of diabetes mellitus caused by hyperglycemia and hypoxia due to metabolic imbalance. Under hypoxic conditions, TTR interacts with and upregulates GRP78 and acts as a trigger of apoptosis of hRECs, which leads to the suppression of neovascularization [21,179]. Under hyperglycemic conditions, TTR represses angiogenesis by inhibiting the proliferation of hRECs through the tyrosine protein kinase receptor 2 (Tie 2) signaling pathway [240]. TTR also suppresses the proliferation of hRECs by long noncoding RNA (lncRNA) MEG3 in the miR-223-3p/FBXW7/Notch1 signaling pathway [225,241]. Therefore, TTR regulates lncRNAs to repress vascular leakage in the retina [226,241]. TTR is also able to regulate and interact with vascular endothelial growth factor [240,242].

Neovascularisation plays an important role in wound healing and atherosclerosis [239,243]. HDL transports miR-223 and other miRNAs [221,224], and signaling of multiple noncoding RNAs is involved in the progression of atherosclerosis [244]. Some lncRNAs are involved in VSMCs phenotype switching. For example, MALAT1/miR-204/SMAD4 regulates osteoblastic differentiation of human aortic valve interstitial cells in CAVD, and TUG1/miR-204-5p promotes osteoblastic differentiation by upregulating Runx2 in aortic valve calcification [244]. lncMEG3 is one of the lncRNAs responsible for pathological cardiac remodeling in atherosclerosis and ECM reconstruction [245], and MEG3 was shown to be downregulated in CAD [246]. The MEG3/miR-223/NLRP3 inflammasome pathway has been shown to enhance inflammatory cell death (pyroptosis) in human aortic endothelial cells [247]. It is of interest to determine whether TTR (directly or indirectly through HDL) regulates the MEG3/miR-223-3p/FBXW7/Notch1 signaling pathway in atherosclerosis.

## 14. TTR at the Crossroads of Vascular Calcification and Bone Mineralization

VC and improper bone calcification, which develop with age and are due to improper signaling, are linked to each other, and there is a negative correlation between pathological calcification and bone density [170]. Vitamin K regulates bone remodeling by promoting the transition of osteoblasts to osteocytes and by restricting osteoclastogenesis [248]. Vitamin-K-dependent γ-carboxylation of glutamate is critical for bone metabolism and for plasma coagulation [248,249]. Proper functioning of the coagulation processes, i.e., formation of the fibrin clot, depends on plasma Ca^2+^. Vitamin K plays stimulatory roles in the coagulation system and bone health by increasing the Ca^2+^-binding capacities of many relevant proteins [249]. Importantly, anti-inflammatory effects of vitamin K and enhancing the activity of matrix Gla proteins prevent mineral vascular deposition, which otherwise leads to calcification/atherosclerosis [250]. γ-Carboxylation of glutamic acid residue 42 of TTR was detected in the CSF in patients with moya-moya disease (MMD) [158]. Stenosis of the large intracranial arteries and proximal extensive vascularization (aberrant angiogenesis) are observed in MMD [251]. MMD is a complex cerebrovascular disease characterized by fibrocellular thickening of the intima, aberrant angiogenesis, matrix accumulation, and improper proliferation of endothelial cells and/or VSMCs [251]. Considering the role of TTR in the regulation of neovascularization in DR, dysregulation of similar regulatory pathways may contribute to MMD pathogenesis. TTR has been included in a group of proteins that have a Gla domain (https://pfam.xfam.org/family/Gla, accessed on 18 May 2021), which confirms the role of TTR at the crossroads of vascular calcification and bone mineralization.

## 15. Concluding Remarks

The native tetrameric form of TTR is a protective factor against oxidative stress. TTR is involved in ROS balance, ECM remodeling, autophagy, apoptosis, reverse HDL cholesterol transport, proliferation, and angiogenesis under physiological conditions and in pathological disorders or stress-induced insults.

The formation of TTR amyloid is induced by oxidative modification, aging, mutation, metal ions (including Ca^2+^), plasmin, and negatively charged polymers. The factors that compromise structural stability and lead to amyloid formation upon dysregulation may be responsible for improper/mislocated induction of TTR and result in cytotoxic TTR amyloid.

The contribution of TTR to cardiovascular and osteoarticular diseases is associated with the formation of TTR amyloid and calcification in the vascular and ligament tissues. Low levels of TTR in the plasma are observed in CVDs and the majority of osteoarticular disorders. It is difficult to determine whether changes in the processes or TTR levels correspond to a cause or a consequence of amyloid formation and whether adverse effects observed in amyloid-induced diseases are a consequence of amyloid overload or a loss of the protective functions of TTR.

Unaggregated/native and aggregated/amyloid TTR forms are interconnected in the following loops (Figure 1).

Vicious cycle 1—oxidative stress: Oxidative modifications lead to TTR destabilization and pathological amyloid, which increases oxidative stress.

Properly folded TTR is a factor that suppresses oxidative stress, inhibits intracellular Ca^2+^ influx, ROS production, membrane permeabilization, apoptosis, and autophagy, and promotes the assembly of oligomeric proteins into larger, less toxic aggregates.

Vicious cycle 2—Ca^2+^: TTR amyloid is formed in situ in response to high Ca^2+^ concentration, which, in turn, promotes TTR destabilization and amyloid deposition, which entraps more Ca^2+^.

Vicious cycle 3—inflammation: Plasmin or other factors induce the formation of TTR amyloid. Amyloid deposits cause plasmin activation and induce inflammation, which, in turn, promotes amyloid formation.

Vicious cycle 4—lipids: Cholesterol and anionic phospholipids bind TTR and promote TTR aggregation. On the other hand, aggregated TTR alters membrane fluidity and induces cytotoxic effects, upregulating TTR aggregation.

## Figures and Tables

**Table 1:** Osteoarticular diseases involving TTR.

Disease	TTR	Selected Effects	Other Factors/Conditions Involved	References
Rheumatoid arthritis	deregulation in plasma, different post-translational modification of TTR cysteine residue 10	inflammation of the synovial membrane	gelsolin, angiotensinogen, lipopolysaccharide-binding protein, protein S100-A9	[129,130]
Juvenile idiopathic arthritis	upregulation in plasma and synovial fluid, oxidized and aggregated TTR forms	chronic autoimmune disorder	innate immunity	[132]
Osteoarthritis	TTR proteolysis, reduced levels of the truncated form, deposition of TTR amyloid in articular cartilage	changed expression of catabolic and inflammatory genes, hypoxia		[126,133,134]
Osteoporosis	low plasma levels	reduced bone mineral density	type 2 diabetes mellitus	[135,136]

**Table 2:** Vascular diseases involving TTR.

Disease	TTR	Selected Symptoms/Effects	Other Factors/Conditions Involved	References
Cardiovascular disease	negative correlation, low plasma levels, positive correlation with the cysteinylated form of TTR	increased mortality, angiographic severity (coronary artery stenosis), vascular stiffness, arterial blockage by atherosclerotic plaque, acute coronary syndrome, mortality	type 2 diabetes mellitus acute coronary syndrome, oxidative stress and changes in the levels of proteins involved in blood coagulation, iron homeostasis, anti-oxidant and immune response, cell-matrix adhesion, response to Ca^2+^, plasmin and thrombin inhibition, HDL remodeling	[149,150,151,154]
Calcific aortic valve disease	amyloid deposits	calcified aortic valve	differentially expressed molecules of transcriptome, proteome, and miRNA, protein–protein interaction network, amyloid β precursor protein	[155]
ATTR cardiac amyloidosis	amyloid deposits, cardiac amyloid infiltration, lowered plasma levels	heart failure with preserved ejection fraction, aortic stenosis, left ventricular wall thickness	increased cardiomyocyte stiffness related to abnormal Ca^2+^ homeostasis preceded by osteoarticular disorders	[146,147,156]
Thromboembolism	upregulated plasma level	elevated thrombin generation and hypofibrinolytic state	reduced DJ-1 activity	[157]
Moya-moya disease	γ-carboxylation of glutamic acid residue 42	extensive vascularization, stenosis		[158]

## Data Availability

No new data were created or analyzed in this study. Data sharing is not applicable to this article.

## Abbreviations

ACS	Acute coronary syndrome
RAGE	Advanced glycation end products
ATTRwt	Aggregated wild type TTR
AD	Alzheimer’s disease
Aβ	Amyloid β
APP	Amyloid β precursor protein
ApoA1	Apolipoprotein A1
ATTR CA	ATTR cardiac amyloidosis
CAVD	Calcific aortic valve disease
CNPs	Calcifying nanoparticles
CA	Cardiac amyloidosis
CVD	Cardiovascular disease
CSF	Cerebrospinal fluid
CAD	Coronary artery disease
DR	Diabetic retinopathy
ECM	Extracellular matrix
FAP	Familial amyloidotic polyneuropathy
HF	Heart failure
HFpEF	Heart failure with preserved ejection fraction
HDL	High density lipoprotein
HMW	High molecular weight
HCy	Homocysteine
hRECs	Human retinal endothelial cells
AL	Immunoglobulin light chain amyloid
JIA	Juvenile idiopathic arthritis
lncRNA	Long noncoding RNA
MMP	Matrix metalloproteinases
TFAM	Mitochondrial transcription factor A
MMD	Moya-moya disease
MPO	Myeloperoxidase
NPY	Neuropeptide Y
OA	Osteoarthritis
PILP	Polymer-induced liquid phase
ROS	Reactive oxygen species
RA	Rheumatoid arthritis
RBP	Retinol-binding protein
SSA	Senile systemic amyloidosis
SAA	Serum amyloid A
TUDCA	Tauroursodeoxycholic acid
tPA	Tissue-type plasminogen activator
T2DM	Type 2 diabetes mellitus
UPR	Unfolded protein response
PAM	Peptidylglycine α-amidating monooxygenase
VC	Vascular calcification
VSMCs	Vascular smooth muscle cells
